# *HyPRP1* performs a role in negatively regulating cotton resistance to *V. dahliae* via the thickening of cell walls and ROS accumulation

**DOI:** 10.1186/s12870-018-1565-1

**Published:** 2018-12-07

**Authors:** Jun Yang, Yan Zhang, Xingfen Wang, Weiqiao Wang, Zhikun Li, Jinhua Wu, Guoning Wang, Liqiang Wu, Guiyin Zhang, Zhiying Ma

**Affiliations:** North China Key Laboratory for Crop Germplasm Resources of Education Ministry, Hebei Agricultural University, Baoding, 071001 China

**Keywords:** Cotton, Hybrid proline-rich protein (HyPRP), *Verticillium dahliae*, Cell wall protein, Reactive oxygen species (ROS), Virus-induced gene silencing (VIGS)

## Abstract

**Background:**

Developing tolerant cultivars by incorporating resistant genes is regarded as a potential strategy for controlling Verticillium wilt that causes severe losses in the yield and fiber quality of cotton.

**Results:**

Here, we identified the gene *GbHyPRP1* in *Gossypium barbadense*, which encodes a protein containing both proline-rich repetitive and Pollen Ole e I domains. GbHyPRP1 is located in the cell wall. The transcription of this gene mainly occurs in cotton roots and stems, and is drastically down-regulated upon infection with *Verticillium dahliae*. Silencing *HyPRP1* dramatically enhanced cotton resistance to *V. dahliae*. Over-expression of *HyPRP1* significantly compromised the resistance of transgenic *Arabidopsis* plants to *V. dahliae*. The *GbHyPRP1* promoter region contained several putative phytohormone-responsive elements, of which SA was associated with gene down-regulation. We compared the mRNA expression patterns of *HyPRP1*-silenced plants and the control at the global level by RNA-Seq. A total of 1735 unique genes exhibited significant differential expression. Of these, 79 DEGs involved in cell wall biogenesis and 43 DEGs associated with the production of ROS were identified. Further, we observed a dramatic thickening of interfascicular fibers and vessel walls and an increase in lignin in the *HyPRP1*-silenced cotton plants compared with the control after inoculation with *V. dahliae*. Additionally, silencing of *HyPRP1* markedly enhanced ROS accumulation in the root tips of cotton inoculated with *V. dahliae*.

**Conclusions:**

Taken together, our results suggest that *HyPRP1* performs a role in the negative regulation of cotton resistance to *V. dahliae* via the thickening of cell walls and ROS accumulation.

**Electronic supplementary material:**

The online version of this article (10.1186/s12870-018-1565-1) contains supplementary material, which is available to authorized users.

## Background

The plant cell wall contains a large set of structural proteins that are involved in defense response. Cell wall proteins are unusually rich in one or two amino acids and contain highly repetitive sequence domains. Currently, much is known about their sequence information, but there is little direct evidence of the functions of these proteins [1]. Hybrid proline-rich proteins (HyPRPs) form a subgroup of putative plant cell wall glycoproteins enriched in proline. HyPRPs are composed of three different domains: a hydrophobic signal peptide, a repetitive proline-rich domain in the N-terminus, and a hydrophobic C-terminal domain, not specifically rich in proline or glycine but containing cysteine. Based on the abundance and specific distribution of cysteine in this C-terminal domain, the HyPRPs are subdivided into A and B groups. In group A, four or six cysteine residues are present in a specific pattern (-CXXC-C-C-C-C-), while group B contains eight cysteine residues (termed the eight-cysteine motif, 8CM) in a specific order (-C-C-CC-CXC-C-C-) in the C-terminal domain [2]. Multiple studies have indicated that group B HyPRPs play various functional roles in specific developmental stages and in response to biotic and abiotic stresses. For example, *CaHyPRP1* performs distinct dual roles as a negative regulator of basal defense and in the positive regulation of cell death in *Capsicum annuum* against *Xanthomonas campestris* [3]. *GmHyPRP* is involved in triggering the soybean resistance response to *Phakopsora pachyrhizi* [4]. The HyPRP gene *EARLI1* (Early Arabidopsis Aluminum Induced 1) is induced in *Arabidopsis* by low temperature and salt stress [5]. *GhHyPRP4* has been reported to take part in the cold stress response of *Gossypium hirsutum* [6]. Overexpression of *CcHyPRP* from *Cajanus cajan* increased resistance to multiple abiotic stresses in yeast and *Arabidopsis* [7]. However, the roles of group A HyPRPs in plant development and defense against pathogen attacks remain unclear, in contrast to the relatively better characterized proteins of group B. As of now, only one group A protein, PvPRP1, has been researched extensively. *PvPRP1* can be down-reglated by *Colletotrichum lindemuthianum* and up-regulated by wounding in the hypocotyls of French bean (*Phaseolus vulgaris*) [8]. The down-regulated expression of *PvPRP1* in response to fungal infection is due to mRNA destabilization through the binding of PRP-BP (*Pv*PRP1 mRNA binding protein) to a 27-nucleotide U-rich domain in the 3′ untranslated region of *Pv*PRP1 mRNA [9].

*Verticillium dahliae* Kleb is a soil-borne pathogenic fungus capable of causing vascular wilt disease in cotton (*Gossypium* spp.). In most cotton-growing areas, Verticillium wilt has become the most important disease of cotton [10]. Unfortunately, currently available fungicides are not effective in protecting cotton from this vascular disease infection. Therefore, developing tolerant cotton cultivars by incorporating genes from resistant germplasm is now regarded as the most effective strategy for controlling this disease. Genetic dissection of Verticillium wilt resistance at the molecular level, as mediated by the relevant genes, will enhance our ability to utilize the existing germplasm to reduce cotton yield losses [11].

In recent years, high-throughput technology has been used to systematically monitor expression profiles and screen a wide spectrum of differentially expressed genes/proteins in cotton inoculated with *V. dahliae* with the goal of ultimately using genetic engineering to breed resistant cultivars. A total of 188 differentially expressed proteins were identified in the roots of *Gossypium barbadense* upon infection with *V. dahliae* based on comparative proteomics analysis [12]. Moreover, 3442 unigenes related to defense responses against *V. dahliae* were identified in *G. barbadense* cv. 7124 using RNA-Seq [13]. In addition, a total of 3027 Veticillium-resistance unigenes were identified from a full-length cDNA library of *G. barbadense* cv Pima90–53 [14]. These comprehensive gene and protein expression data provide helpful molecular information. However, deeper insights into understanding the defense mechanisms of cotton in response to *V. dahliae* are needed.

Previously, we obtained a substantial number of transcript sequences from *G. barbadense* related to defense responses against *V. dahliae* [14]. Of these, a cDNA clone that encodes a group A HyPRP protein, designated as *GbHyPRP1*, whose expression was significantly down-regulated in cotton after *V. dahliae* inoculation. In the present study, *GbHyPRP1* and its homologous genes were cloned from other *G. hirsutum* cultivars. The transcriptional expression of *HyPRP1* was investigated in different tissues and in response to treatment with different hormones and *V. dahliae.* A potential role of *HyPRP1* in negatively regulating plant resistance to *V. dahliae* was examined by overexpression in *Arabidopsis* and by virus-induced gene silencing (VIGS) in cotton. We applied transcriptomic analysis to systematacially explore the molecular mechanisms underlying the *HyPRP1*-mediated cotton defensive response to *V. dahliae*. The important function of *HyPRP1* involved in cotton resistance to *V. dahliae* via the thickening of the cell wall and ROS accumulation was proved.

## Results

### GbHyPRP1 is a cell wall protein down-regulated by *V. dahliae* challenge

**Fig. 1:**
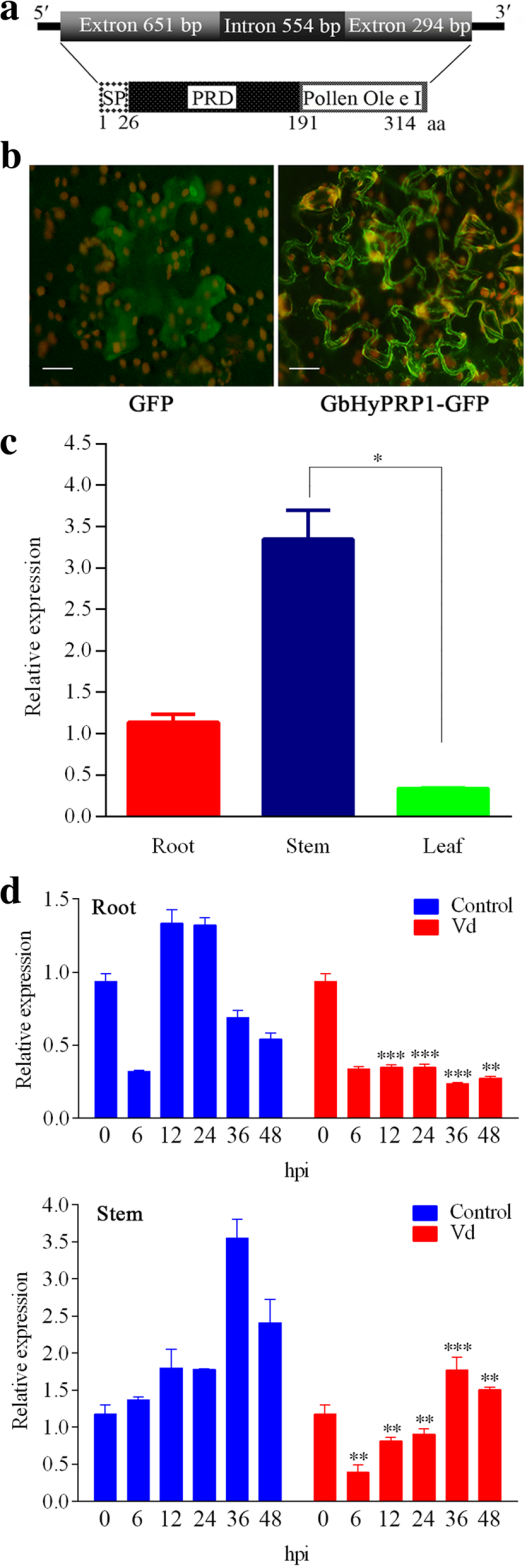
Characterization of *GbHyPRP1*. **a** Schematic structure of *GbHyPRP1*. **b** Subcellular localization of GFP alone or GbHyPRP1-GFP fusion in tobacco leaves transiently transformed by *Agrobacterium* infiltration. The green fluorescence were monitored using a confocal laser scanning microscope. Bars = 10 μm. **c** Tissue-specific expression of *GbHyPRP1* by qPCR. Two-week-old *G. barbadense* Pima90–53 plants were used for sampling. The values were normalized to gene *PP2A1*. The significant differences in expression level of *GbHyPRP1* in different tissues were evaluated by the non-parametric Kruskal-Wallis test followed by Dunn’s multiple comparisons test. The bar represents mean ± SE from three biological replicates (*P < 0.05). **d** Transcriptional analysis of *GbHyPRP1* were measured in response to *V. dahliae* (Vd) compared to the control by qPCR. The roots and stems of two-week-old Vd-exposed Pima90–53 seedlings and control (water-treated) were collected. The values were normalized to gene *PP2A1.* The bar represents mean ± SE from three biological replicates. Sidak’s multiple comparisons test demonstrated that there were significant differences (**P < 0.01, ***P < 0.001) between Vd and control at 0, 6, 12, 24, 36 and 48 h post-inoculation (hpi)

Previously, we isolated a full-length cDNA clone from a full-length cDNA library of *G. barbadense* Pima90–53 challenged with *V. dahliae* [14]. The cDNA clone has a 5′ untranslated region (5′ UTR) of 41 bp, 3′ UTR of 186 bp with a polyA tail and an open reading frame (ORF) of 945 bp that potentially encodes a 314-amino acid protein. The protein consists of a 26-residue signal peptide (SP) sequence with an initial ATG codon, a 165-residue proline-rich domain (PRD) (containing a basic histidine-rich domain) at the N-terminus and a 123-residue hydrophobic C-terminus Pollen Ole e I domain (cysteine-containing domain) (Fig. 1a). Based on the sequence features and a homology search, the protein belongs to group A HyPRPs, designated as *GbHyPRP1* (GenBank accession number KP162172). GbHyPRP1 has a predicted molecular weight of ~ 33.59 kDa with a theoretical pI of 9.97. The alignment results showed that GbHyPRP1 from *G. barbadense* shared a high sequence identity with the other GhHyPRP1 proteins from six cultivars of *G. hirsutum* (Additional file 1: Figure S1).

A GFP gene fused to the C-terminal end of *GbHyPRP1* under the control of the constitutive CaMV 35S promoter was successfully transformed into tobacco epidermal cells and transiently expressed. The control GFPs appeared to be distributed throughout the whole cell, which indicated that GFPs were localized to plasma membrane and cytoplasm. By contrast, the GFP signal indicated that the fusion HyPRP1-GFP proteins were localized to the cell periphery (Fig. 1b). In addition, GbHyPRP1 belongs to group A HyPRPs (Fig. 1a), which form a subgroup of plant cell wall glycoproteins enriched in proline. Thus, we inferred that HyPRP1 localized to the cell wall.

As shown in Fig. 1c, *GbHyPRP1* was expressed in all tested parts of the cotton plants, with a significantly higher expression level in the stem compared with the leaf. In addition, the expression profiles of *GbHyPRP1* in response to the highly aggressive defoliating *V. dahliae* strain were examined in infected *G. barbadense* roots and stems. The qPCR (real-time quantitative PCR) analysis showed that the expression level of *GbHyPRP1* significantly decreased in either stems or roots after inoculation with *V. dahliae* (Fig. 1d). These data indicate that *GbHyPRP1* is involved in the cotton-*V. dahliae* interaction.

### *V. dahliae*-responsive expression of *HyPRP1* exhibits the same trend in *G. hirsutum* as in *G. barbadense*

**Fig. 2:**
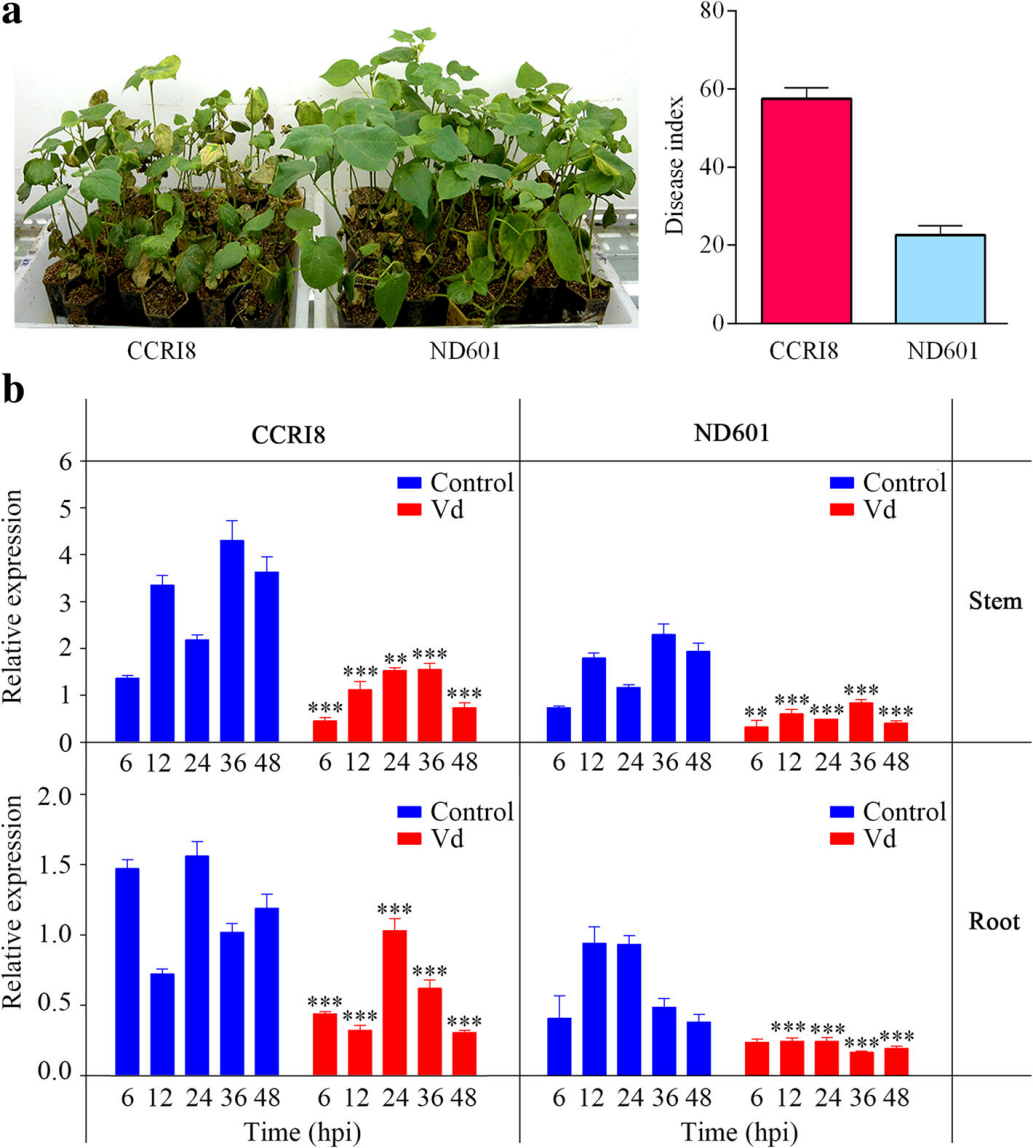
Time-related changes in the expression of *HyPRP1* in response to *V. dahliae* (Vd) in *G. hirsutum*.**a** ND601 showed higher tolerence to *V. dahliae* (left panel) with lower disease index (right panel) compared to CCRI8 at 14 dpi. Two-week-old seedlings were dip-infected with the *V. dahliae* spores. The disease indices were presented means ± SE from three biological replications with at least 15 plants per replication. **b**, The expression of *HyPRP1* changed to a lower degree both in stems and roots of tolerent ND601 and susceptible CCRI8 after infection using *V. dahliae* comparing to the control at the same hpi. Meanwhile, the relative expression of *HyPRP1* displayed more lower both in stems and roots of tolerent ND601 comparing to the susceptible CCRI8 after infection using *V. dahliae* at the same hpi. The relative gene expression was calculated using the comparative 2^-*ΔΔCt*^ method with *PP2A1* as endogenous control gene. Values are shown as the mean ± SE of three biological replicates. Sidak’s multiple comparisons test demonstrated that there were significant differences (**P < 0.01, ***P < 0.001) between Vd and control

We further determined the *V. dahliae*-responsive expression of *HyPRP1* in *G. hirsutum* using a resistant cv. ND601(DI = 22.63 ± 2.28) and a susceptible cv. CCRI8 (DI = 57.59 ± 2.76) [15] inoculated with *V. dahliae* (Fig. 2)a*.* The results showed that the expression of *HyPRP1* dramatically decreased in both resistant and susceptible cotton stems and roots after inoculation with *V. dahliae* compared to the control (Fig. 2b). Moreover, the expression of *HyPRP1* in the resistant cv. ND601 was significantly lower compared to the susceptible cv. CCRI8 (Fig. 2b). These results ulteriorly indicated that *HyPRP1* is a negative regulator involved in the cotton-*V. dahliae* interaction.

### Silencing of *HyPRP1* enhances cotton resistance to Verticillium wilt

**Fig. 3:**
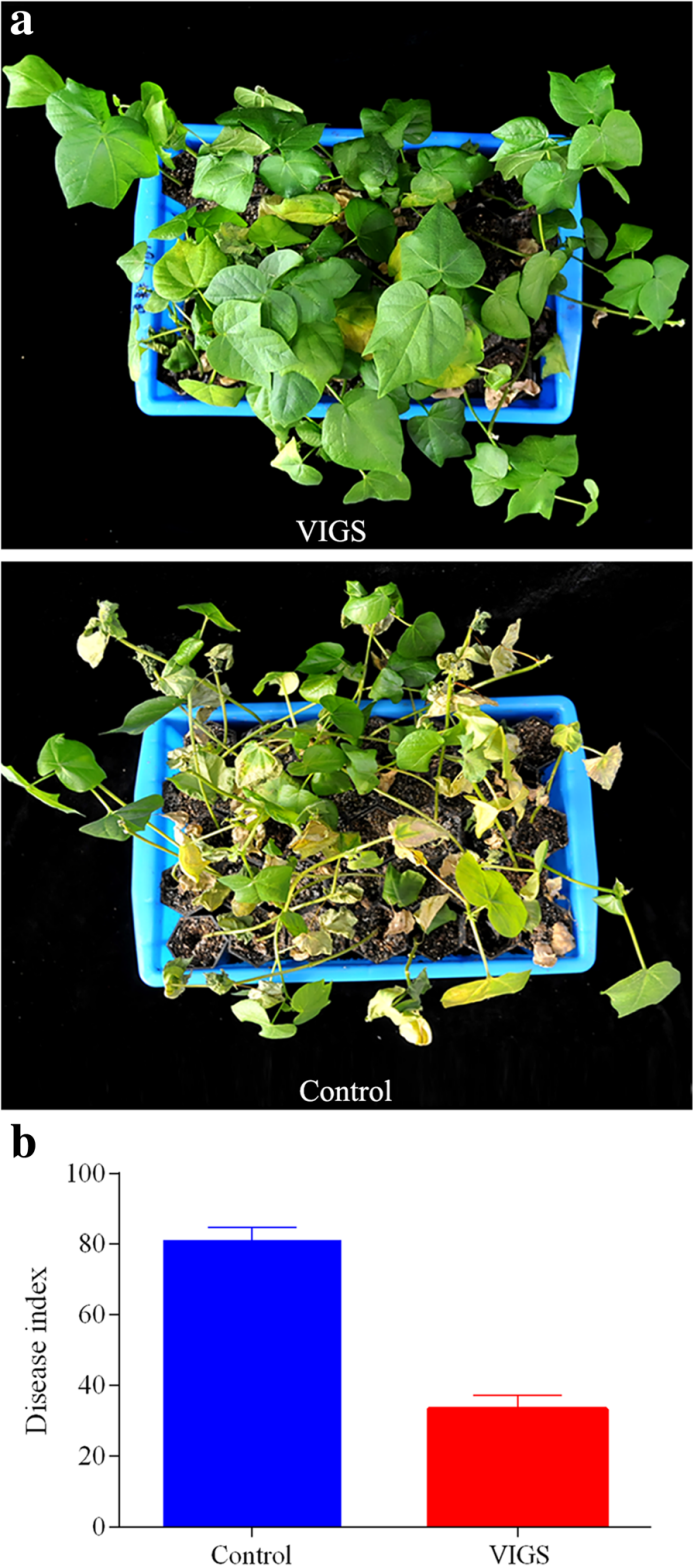
Silencing of *HyPRP1* enhanced cotton plant resistance to Verticillium wilt. **a** Disease symptoms of *HyPRP1* VIGS CCRI8 plants after inoculation with *V. dahliae* strain Linxi2–1. Control plants were infiltrated with *Agrobacterium* carrying a VIGS empty vector. Photographs were taken at 15 dpi. **b** The disease index was measured at 15 dpi. Error bars represent SE of three biological replicates with at least 35 plants per replication

We further tested whether *HyPRP1* was required for cotton resistance to Verticillium wilt using VIGS, which is frequently employed as a reverse genetics technique. At approximately two weeks post-infiltration, marker gene *CLA1*-VIGS plants started to display the albino phenotype in the true leaves (Additional file 2: Figure S2A). At the same time, the expression of *HyPRP1* in susceptible CCRI8 was not detected using semi-RT-PCR, indicating that *HyPRP1* had been silenced (Additional file 2: Figure S2B). HyPRP1-silenced plants were used for Verticillium inoculation. At 15 days post inoculation (dpi), less chlorosis and fewer wilting leaves were observed in VIGS plants compared to the control (Fig. 3a). The DI of VIGS plants (32.67 ± 1.96) was significantly lower than that of the control (81.00 ± 2.13), i.e., susceptible CCRI8 became tolerent (Fig. 3b). VIGS assays indicated that silencing of *HyPRP1* significantly enhances cotton resistance to Verticillium wilt.

### Over-expression of *GbHyPRP1* compromises *Arabidopsis* resistance to Verticillium wilt

**Fig. 4:**
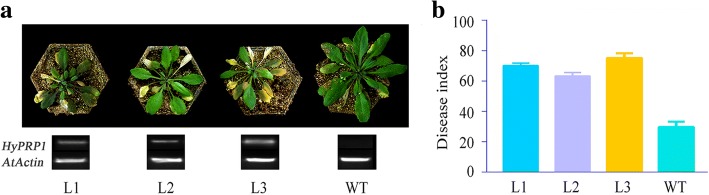
Over-expression of *GbHyPRP1* compromised *Arabidopsis* resistance to Verticillium wilt. **a** Phenotype comparison of transgenic lines (L1, L2 and L3) and the WT inoculated with *V. dahliae*. Photographs were taken at 15 dpi. Expression of *GbHyPRP1* was confirmed using semi-qRT-PCR. The *AtActin* gene was used as a control. **b** The disease index was measured at 15 dpi. Error bars represent the SE of three biological replicates with at least 20 plants per replication

To further determine the involvement of *GbHyPRP1* in Verticillium wilt resistance, plant over-expression vectors containing the *GbHyPRP1* ORF driven by a constitutive 35S promoter were constructed and transformed (*Agrobacterium*-mediated) into *Arabidopsis thaliana*. Three lines, L1, L2 and L3, were used for further analyses. After 15 dpi with *V. dahliae*, all the transgenic lines exhibited more wilting and etiolation compared to WT (wild type) (Fig. 4a). The average DI of transgenic plants, i.e., 69.5 (classifed as susceptable), was significantly higher than that of the control (29.8; classified as tolerant) (Fig. 4b). These results suggest that over-expression of *GbHyPRP1* compromised *Arabidopsis* resistance to Verticillium wilt.

### *GbHyPRP1* contains hormone elements in the promoter and is down-regulated by SA

**Fig. 5:**
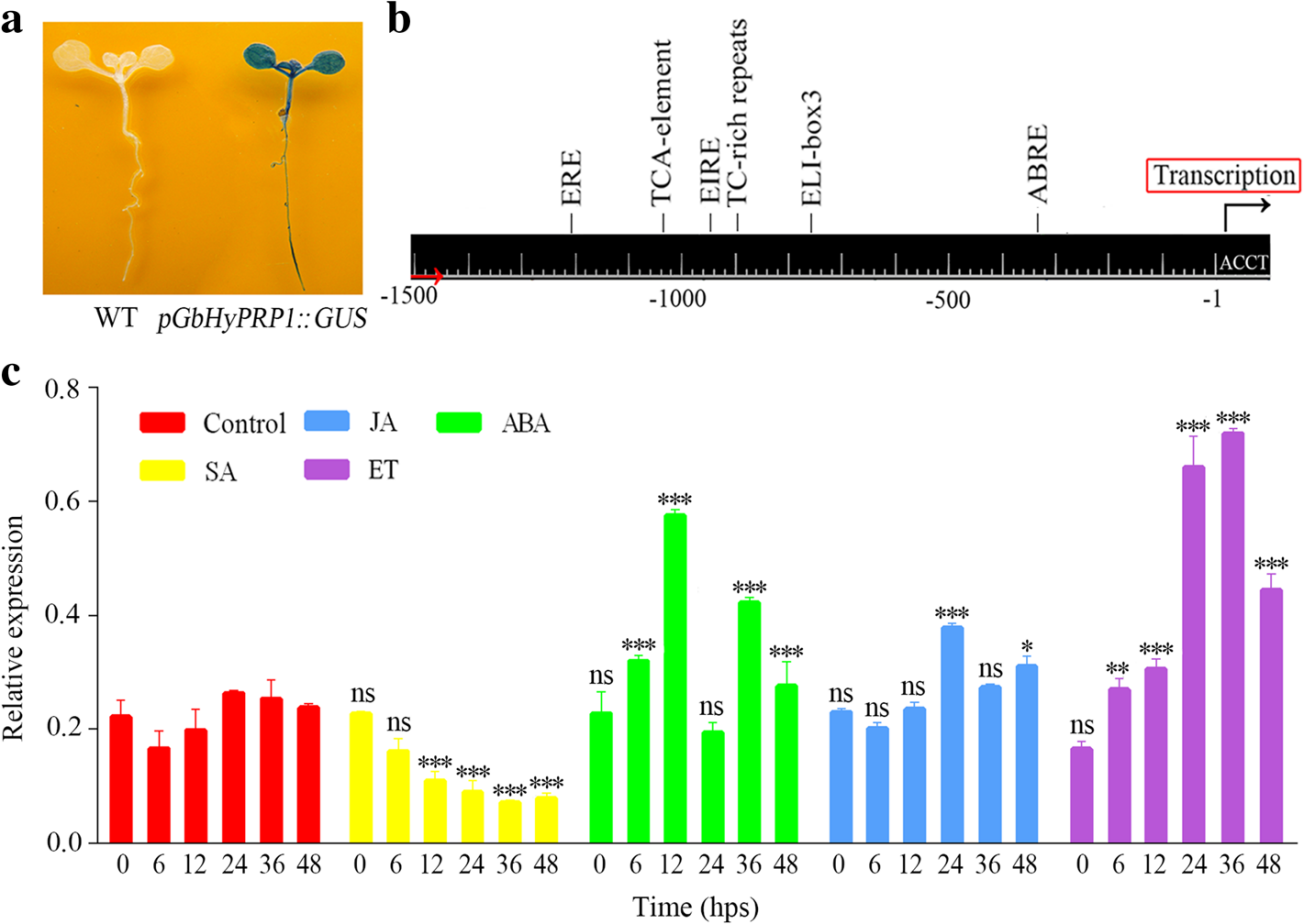
Properties of p*GbHyPRP1*. **a** Histochemical analysis of GUS activity in *Arabidopsis* expressing the p*GbHyPRP1*-GUS chimeric gene. GUS activity was present in whole 7-day-old seedlings. **b** Location of *cis*-regulatory elements involved in hormone- and elicitor-responsive elements found in p*GbHyPRP1*. The conserved fungus-responsive elements ELI-box3 (− 216) were present in the promoter region of WRKY in the *Populus*, while the EIRE (elicitor responsive element) (−879) are the binding site of WRKY and required for elicitor responsiveness in the promoter of PR (pathogenesis-related protein) in the parsley [64, 65]. TC-rich repeat elements (− 502) are putatively involved in plant defense and stress response. Furthermore, ABRE (ABA-responsive element) (− 292), TCA-element and ERE (ethylene responsive element) (− 1128) have been reported in the upstream region of many genes that show regulated expression in response to ABA, SA and Et, respectively [26–28]. **c** Expression profiles of *GbHyPRP1* in leaves of 15-day-old Pima90–53 seedlings subjected to SA, ABA, JA and ET. Control indicates water treatment. Three plants at each time point were sampled and analysed. Values are presented as the means ± SE in three independent experiments. Asterisks represent significant differences with respect to the control as determined by Tukey’s multiple comparisons test (*P < 0.05, ** *P* < 0.01; *** *P* < 0.001; ns = not significant)

The upstream region of *GbHyPRP1,* named p*GbHyPRP1,* was 1431 bp in length based on sequencing (Additional file 3: Figure S3). p*GbHyPRP1* fused with the reporter gene GUS introduced into *Arabidopsis* plants, and GUS staining confirmed that the promoter of *GbHyPRP1* functioned well with respect to the expression of the reporter gene (Fig. 5a). Promoter search using PLANTCARE indicated that several potentially inducible *cis*-regulatory elements corresponding to hormone, defense and pathogen elicitor responses were found in p*GbHyPRP1* (Fig. 5b). Thus, we investigated the expression profile of *GbHyPRP1* following treatments with plant hormones, including SA (salicylic acid), abscisic acid (ABA), jasmonic acid (JA) and ethylene (ET). The results indicated that the expression of *GbHyPRP1* was strongly down-regulated by SA at 12, 24, 36 and 48 hps (hours post-spraying), whereas it was significantly up-regulated by ABA, JA and ET at various time points (Fig. 5c). To our surprise, the *HyPRP1* transcript levels in the SA-treated plants were quite similar to those in the *V. dahliae*-inoculated cotton seedlings (Figs. 1d and 2b). These results imply that *HyPRP1* may play roles in cotton resistance to *V. dahliae* through the SA-mediated signaling pathway.

### Transcriptome analysis of VIGS cotton indicated *HyPRP1* influences genes related to cell wall remodeling and ROS balance

To better understand the molecular mechanisms of HyPRP1-mediated cotton defense response to *V. dahliae*, we empoyed a combined approach of VIGS and RNA-Seq to compare the mRNA expression patterns of *HyPRP1*-silenced and control plants at the global level. Transcriptome libraries yielded 56,364,572 to 63,092,290 raw reads. After cleaning and quality checks, 55,741,348 to 62,384,322 high quality reads were obtained, and 79.66–80.87% of these reads were uniquely mapped to *G. hirsutum* L. acc. TM-1 [16], representing more than forty thousand unigenes(RPKM ≥1)(Table 1). Under *V. dahliae* stress, a total of 1735 unique genes exhibited significant differential expression based on VIGS compared to the control, including 816 up-regulated and 919 down-regulated genes (*P*-value ≤ 0.05) (Additional file 4: Data S1).

**Table 1:** Summary of sequencing, read processing, mapping, and differential expression analysis

Sample names & Replicates	Control-12 hpi (C12)		VIGS-12 hpi (V12)	
	C12–1	C12–2	V12–1	V12–2
Raw reads	60,758,592	56,364,572	59,201,676	63,092,290
Clean reads	60,228,094	55,741,348	58,628,694	62,384,322
Q20 (%)	96.12	96.11	96.13	96.20
Q30 (%)	92.44	92.49	92.46	92.58
GC content (%)	44.62	43.65	45.03	45.34
Total mapped	56,294,373 (93.47%)	51,961,420 (93.22%)	54,610,037 (93.15%)	58,335,113 (93.51%)
Uniquely mapped	48,617,647 (80.72%)	45,077,574 (80.87%)	46,702,784 (79.66%)	49,786,940 (79.81%)
Detected genes(RPKM≥1)	46,067	46,695	42,211	43,636

Of these, we identified 79 differentially expressed genes (DEGs) that might be involved in cell wall biogenesis in cotton according to the annotation from the cell wall genomics webserver (https://cellwall.genomics.purdue.edu/intro/index.html) (Table 2). Expansins and xyloglucan-modifying enzymes are usually up-regulated by various stresses and then become involved in cell wall remodeling [17]. Our RNA-Seq results indicated that the expression of six expansin genes (Gh_A10G1374, Gh_A05G2385, Gh_A05G3493, Gh_A13G0050, Gh_D04G1924 and Gh_D05G2650) and seven xyloglucan endotransglucosylase/hydrolase (XEH) genes (Gh_A02G1426, Gh_A11G1910, Gh_D03G0294, Gh_D11G2065, Gh_A11G0455, Gh_D05G1444 and Gh_D02G1371) were significantly up-regulated in *HyPRP1* VIGS cotton seedlings inoculated with *V. dahliae* compared to the control. Additionally, reactive oxygen species (ROS) have been shown to be associated with cotton resistance to fungal pathogens [18]. Here, we found 43 DEGs associated with the production and scavenging of H_2_O_2_ and O_2_^−^, which allows regulation of dynamic changes in ROS levels (Table 3). Thus, our results suggest that HyPRP1 as a cell wall structural protein might have a potential role in cotton resistance to *V. dahliae* infection through remodeling of the cotton cell wall and ROS production.

**Table 2:** DEGs involving in cell wall biogenesis according to the annotation from the cell wall genomics webserver

Stages of cell wall biogenesis	Gene	Cotton ID	Arabidopsis ID	Biological process description	log2FC	padj
1. Pathways of substrate generation	MPG	Gh_D13G1445	AT4G26850	Mannose-1-phosphate guanylyltransferase	1.3226	0.00074702
	MPG	Gh_A04G0114	AT4G26850	Mannose-2-phosphate guanylyltransferase	1.0154	0.0038664
	MPG	Gh_D05G3607	AT4G26850	Mannose-3-phosphate guanylyltransferase	0.87197	0.016364
	PAL2	Gh_D06G0758	AT3G53260	Phenylalanine ammonia-lyase 2	−4.7679	0.031477
	COMT	Gh_D08G2702	AT5G54160	*O*-methyltransferase 1	−2.1454	1.04E-05
2. Polysaccharide synthases and glycosyl transferases	CSLD5	Gh_D12G1289	AT1G02730	Cellulose synthase-like D5	−1.8163	4.86E-06
	CSLD5	Gh_A12G1169			−1.7886	0.00023721
	CSA2	Gh_D05G2313	AT4G39350	Cellulose synthase A2	0.94515	0.0088594
	UGT	Gh_A01G1073	AT5G65550	UDP-Glycosyltransferase superfamily protein	−1.0906	0.0034716
	UGT	Gh_A08G0702	AT5G12890		1.3521	0.0044964
	UGT2	Gh_D02G0230	AT1G05530	UDP-glucosyl transferase 75B2	1.7995	0.04585
	UGT	Gh_A12G0455	AT3G21780	UDP-glucosyl transferase 71B6	1.1842	0.026133
	GT35	Gh_A13G0714	AT3G29320	Glycosyl transferase, family 35	−1.4741	0.0049557
3. Secretion and targeting pathways	DJC24	Gh_A11G1350	AT4G12780	DNA J protein C24	0.93104	0.032233
	DJC24	Gh_D03G0085	AT2G17880		0.83759	0.036168
	DJC75	Gh_D05G2948	AT4G09350	DNA J protein C75	1.28	0.00014472
	DJC75	Gh_A05G2646			1.3309	0.00072978
	DNJ	Gh_D11G2787	AT1G56300	Chaperone DnaJ-domain superfamily protein	−2.2389	1.13E-09
	DNJ	Gh_D12G1290			0.84609	0.048095
	DNJ	Gh_A11G2469			−2.4022	2.03E-10
	DNJ	Gh_A12G1170			1.2513	0.00021456
	CML12	Gh_Sca058336G01	AT2G41100	Calmodulin-like 12	1.1651	0.00085002
	CML12	Gh_A05G1998			0.97104	0.03541
	CML12	Gh_D07G0377			0.9888	0.0122
	CML30	Gh_A11G3105	AT2G15680	Calmodulin-like 30	0.90289	0.019411
	CaBP	Gh_D01G1024	AT1G73630 AT1G21550	Calcium-binding EF-hand family protein	1.1131	0.033647
	CaBP	Gh_D05G2265			1.1609	0.00045229
	CaBP	Gh_A05G2022			1.0194	0.02553
4. Assembly, architecture, and growth	EXP5	Gh_A10G1374	AT3G29030	Expansin A5	1.0678	0.015918
	EXP8	Gh_A05G2385	AT2G40610	Expansin A8	1.1376	0.00071198
	EXP8	Gh_A05G3493			1.2031	0.00047444
	EXP8	Gh_A13G0050			1.0126	0.020419
	EXP8	Gh_D04G1924			1.0878	0.0013803
	EXP8	Gh_D05G2650			1.1075	0.0011793
	EXP11	Gh_A05G1576	AT1G20190	Expansin A11	−1.5462	0.00073074
	XTH6	Gh_A02G1426	AT5G65730	Xyloglucan endotransglucosylase/hydrolase 6	1.0514	0.0026916
	XTH6	Gh_A11G1910			1.7179	0.0045263
	XTH6	Gh_D03G0294			1.3393	0.0049521
	XTH6	Gh_D11G2065			1.3826	4.78E-05
	XTH7	Gh_A11G0455	AT4G37800	Xyloglucan endotransglucosylase/hydrolase 7	1.0837	0.025074
	XTH16	Gh_D05G1444	AT3G23730	Xyloglucan endotransglucosylase/hydrolase 16	1.0522	0.022367
	XTH28	Gh_D02G1371	AT1G14720	Xyloglucan endotransglucosylase/hydrolase 28	0.96782	0.021935
	GH17	Gh_A01G0299	AT2G39640	Glycosyl hydrolase family 17 protein	−2.7529	0.0037067
	GH17	Gh_A05G0191			−1.5162	0.0061888
	GH17	Gh_Sca016465G01			−1.5804	0.023458
	GH9B13	Gh_D03G0779	AT4G02290	Glycosyl hydrolase 9B13	−1.7925	0.0030996
	GH32	Gh_A06G0779	AT3G13790	Glycosyl hydrolases family 32 protein	−1.6456	0.011594
	BG3	Gh_D06G2277	AT3G57240	Beta-1,3-glucanase 3	−2.4743	0.041623
	PLL	Gh_A03G0087	AT5G47500 AT1G65570 AT4G13710	Pectin lyase-like superfamily protein	−3.5536	1.03E-10
	PLL	Gh_A10G1707			−4.0659	0.0010399
	PLL	Gh_D03G1564			−2.7246	7.22E-10
	PLL	Gh_D05G3049			−1.5099	0.011417
	PLL	Gh_D12G2158			−2.0499	0.00090874
	GRP	Gh_D10G1727	AT3G06780	Glycine-rich protein	1.0437	0.036293
	HRGP	Gh_A06G1050	AT3G25690	Hydroxyproline-rich glycoprotein family protein	−1.3346	0.027913
	HRGP	Gh_D11G0769	AT3G02120		−1.742	0.00058494
5. Differentiation and secondary wall formation	GER3	Gh_A05G3949	AT5G20630	Germin 3	−1.8095	0.012068
	TPX1	Gh_D05G1251	AT1G65980	Thioredoxin-dependent peroxidase 1	1.0563	0.029966
	PRX	Gh_A07G0275	AT2G24800	Peroxidase superfamily protein	−1.5209	0.00086829
	PRXR1	Gh_A05G0507	AT2G24800		−2.0256	0.037339
	PRX52	Gh_A09G2334	AT5G05340		1.4635	0.0033052
	PRX53	Gh_D08G2420	AT5G06720		−3.5232	0.0049155
6. Signaling and response mechanisms	PR5	Gh_A01G1376	AT4G38670	Pathogenesis-related thaumatin superfamily protein	1.3177	0.0017125
	PR5	Gh_A03G0347	AT2G28790		1.6781	0.031724
	PR5	Gh_D12G0310	AT4G38670		1.3177	0.0017125
	LTPG	Gh_A08G0720	AT1G55260 AT2G45180	Lipid-transfer protein	0.81073	0.046398
	LTPG	Gh_A08G1543			1.9495	0.022371
	LTPG	Gh_D08G1844			1.2797	0.011976
	LTPG	Gh_A07G0235			0.88365	0.016833
	LYM2	Gh_A12G0303	AT2G17120	Lysm domain GPI-anchored protein 2 precursor	−1.5656	0.040813
	LYM2	Gh_D12G0361			−1.4063	0.0022
7. Others	SKS4	Gh_A06G1309	AT4G22010	SKU5 similar 4	−1.3884	0.046854
	SKS4	Gh_D06G1637			−1.8953	0.0019284
	PMEI	Gh_A08G1555	AT5G20740 AT5G62360	Plant invertase/pectin methylesterase inhibitor superfamily protein	1.3284	0.00058494
	PMEI	Gh_D03G1026			−2.289	3.90E-13
	PMEI	Gh_D05G0356			1.1199	0.0018667
	PMEI	Gh_D08G1863			1.2925	0.00051083
	CIF1	Gh_D10G1801	AT1G47960	Cell wall / vacuolar inhibitor of fructosidase 1	1.1969	0.00078794
	CIF1	Gh_A10G1552			0.85616	0.047869

**Table 3:** DEGs involving in regulating ROS blance

Cotton ID	log2FC	padj	Arabidopsis ID	Biological function
Gh_D05G1251	1.0563	0.029966	AT1G65980	Thioredoxin-dependent peroxidase 1
Gh_D10G1930	−2.4149	6.56E-07	AT3G06730	Thioredoxin z
Gh_A10G1673	−2.3963	0.0013815	AT3G06730	Thioredoxin z
Gh_A05G3692	−1.1174	0.006171	AT1G76760	Thioredoxin Y1
Gh_D11G1225	1.4097	2.15E-05	AT2G30540	Thioredoxin superfamily protein
Gh_D09G1113	−1.432	8.29E-05	AT2G31840	Thioredoxin superfamily protein
Gh_D08G2582	1.5176	0.0029746	AT4G33040	Thioredoxin superfamily protein
Gh_D05G2291	0.96122	0.0058703	AT4G03520	Thioredoxin superfamily protein
Gh_D05G0426	1.1171	0.012918	AT4G33040	Thioredoxin superfamily protein
Gh_A12G0064	1.0842	0.03541	AT2G30540	Thioredoxin superfamily protein
Gh_A11G1072	1.4971	8.01E-06	AT2G30540	Thioredoxin superfamily protein
Gh_A09G1107	−1.1404	0.0022929	AT2G31840	Thioredoxin superfamily protein
Gh_A05G2047	1.0221	0.0026406	AT4G03520	Thioredoxin superfamily protein
Gh_A05G0320	2.1073	0.0079978	AT4G33040	Thioredoxin superfamily protein
Gh_A01G0925	0.93891	0.0080796	AT3G51030	Thioredoxin H-type 1
Gh_D12G1273	−0.85546	0.036125	AT2G47470	Thioredoxin family protein
Gh_D07G2378	−2.2432	0.0089634	AT4G29720	Polyamine oxidase 5
Gh_A08G1751	1.1958	0.0029528	AT5G21105	Plant L-ascorbate oxidase
Gh_A11G0771	0.86508	0.024855	AT1G01820	Peroxin 11c
Gh_D07G0417	0.83262	0.032604	AT3G47430	Peroxin 11B
Gh_A07G0355	1.0949	0.0017285	AT3G47430	Peroxin 11B
Gh_A09G2334	1.4635	0.0033052	AT5G05340	Peroxidase superfamily protein
Gh_A07G0275	−1.5209	0.00086829	AT2G24800	Peroxidase superfamily protein
Gh_A05G0507	−2.0256	0.037339	AT2G24800	Peroxidase superfamily protein
Gh_D08G2420	−3.5232	0.0049155	AT5G06720	Peroxidase 2
Gh_D01G1856	−1.675	0.01222	AT1G77510	PDI-like 1–2
Gh_A05G3724	−1.0492	0.012948	AT1G21750	PDI-like 1–1
Gh_A04G0409	0.88963	0.01631	AT1G17180	Glutathione S-transferase TAU 25
Gh_A04G0830	0.77974	0.047869	AT1G59700	Glutathione S-transferase TAU 16
Gh_D13G0032	−2.0914	0.045894	AT5G01420	Glutaredoxin family protein
Gh_D12G0079	1.7875	0.010834	AT2G47880	Glutaredoxin family protein
Gh_D11G0244	1.1813	0.00090549	AT1G64500	Glutaredoxin family protein
Gh_A11G0230	1.2723	0.0011017	AT1G64500	Glutaredoxin family protein
Gh_A09G1302	−1.7113	0.047546	AT5G03870	Glutaredoxin family protein
Gh_A05G2978	−2.0779	7.21E-07	AT5G40760	Glucose-6-phosphate dehydrogenase 6
Gh_D07G0457	1.2396	0.036439	AT5G51100	Fe superoxide dismutase 2
Gh_A07G0392	−1.4372	0.0035132	AT5G51100	Fe superoxide dismutase 2
Gh_D11G1719	−0.94414	0.01944	AT1G65930	Cytosolic NADP+ − dependent isocitrate dehydrogenase
Gh_A05G0722	−2.2649	0.0088139	AT2G28190	Copper/zinc superoxide dismutase 2
Gh_D03G0021	1.3352	0.023812	AT4G35090	Catalase 2
Gh_A13G0827	1.1257	0.0022698	AT1G08570	Atypical CYS HIS rich thioredoxin 4
Gh_D10G2577	1.2429	0.034842	AT5G65110	Acyl-coa oxidase 2

### Silencing of *HyPRP1* causes a drastic increase in cell wall thickness and the lignin content

**Fig. 6:**
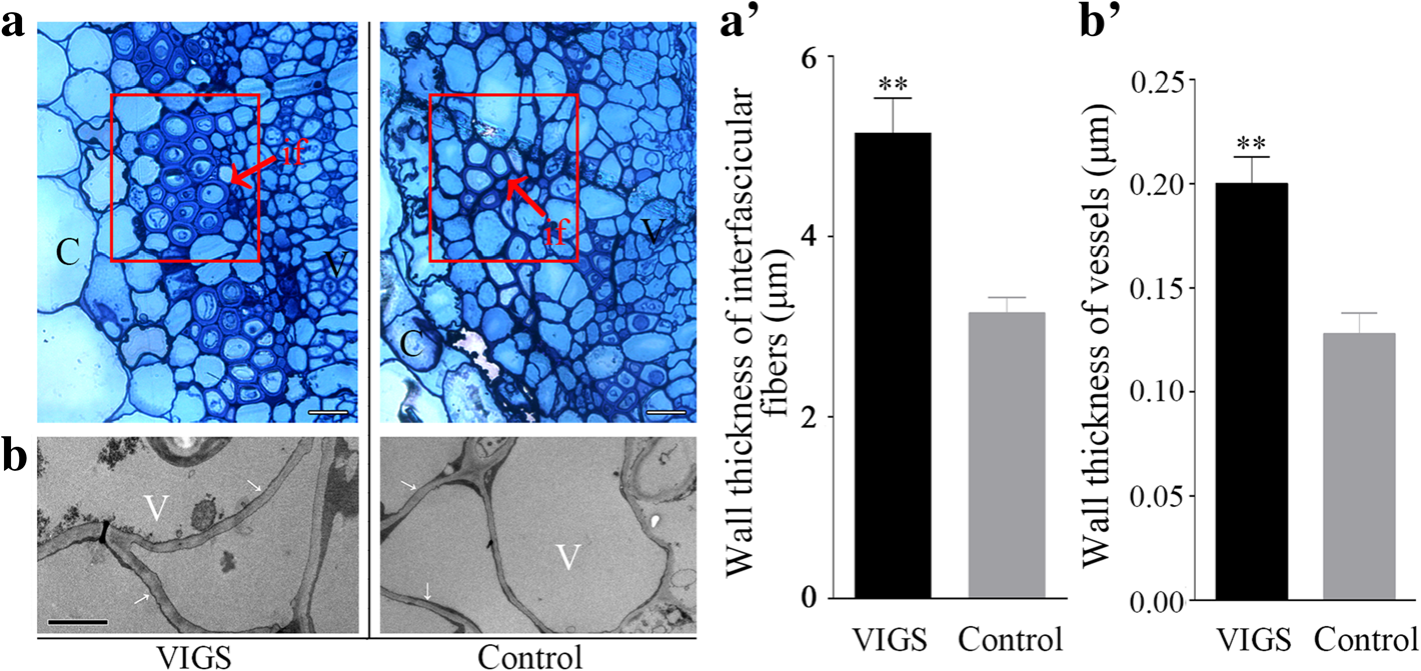
Cell wall thickening in interfascicular fibers and vessels in *HyPRP1*-silenced cotton plants at 14 days post inoculation with *V. dahliae*. **a** Cross-sections of the vascular bundle region of the control and *HyPRP1*-silenced plants. Scale bar = 10 μm. c, cortex; if, interfascicular fiber; v, vessel. **a′**, Measurement and statistical analysis of cell wall thickness in interfascicular fibers. **b**, Transmission electron micrographs of vessel walls of the control and *HyPRP1*-silenced plants. Scale bar = 2 μm. **b′**, Measurement and statistical analysis of cell wall thickness in vessels. 10-day-old seedlings with two fully expanded cotyledons were used for VIGS. After two weeks later *HyPRP1*-silenced plants were inoculated with *V. dahliae*. For each treatment, six separate plants were examined. 10 or more cells were measured for each plant. Data shown are means ± SE of three independent experiments. Asterisks indicate a statistically significant difference according to the non-parametric Mann Whitney test (**P < 0.01)

**Fig. 7:**
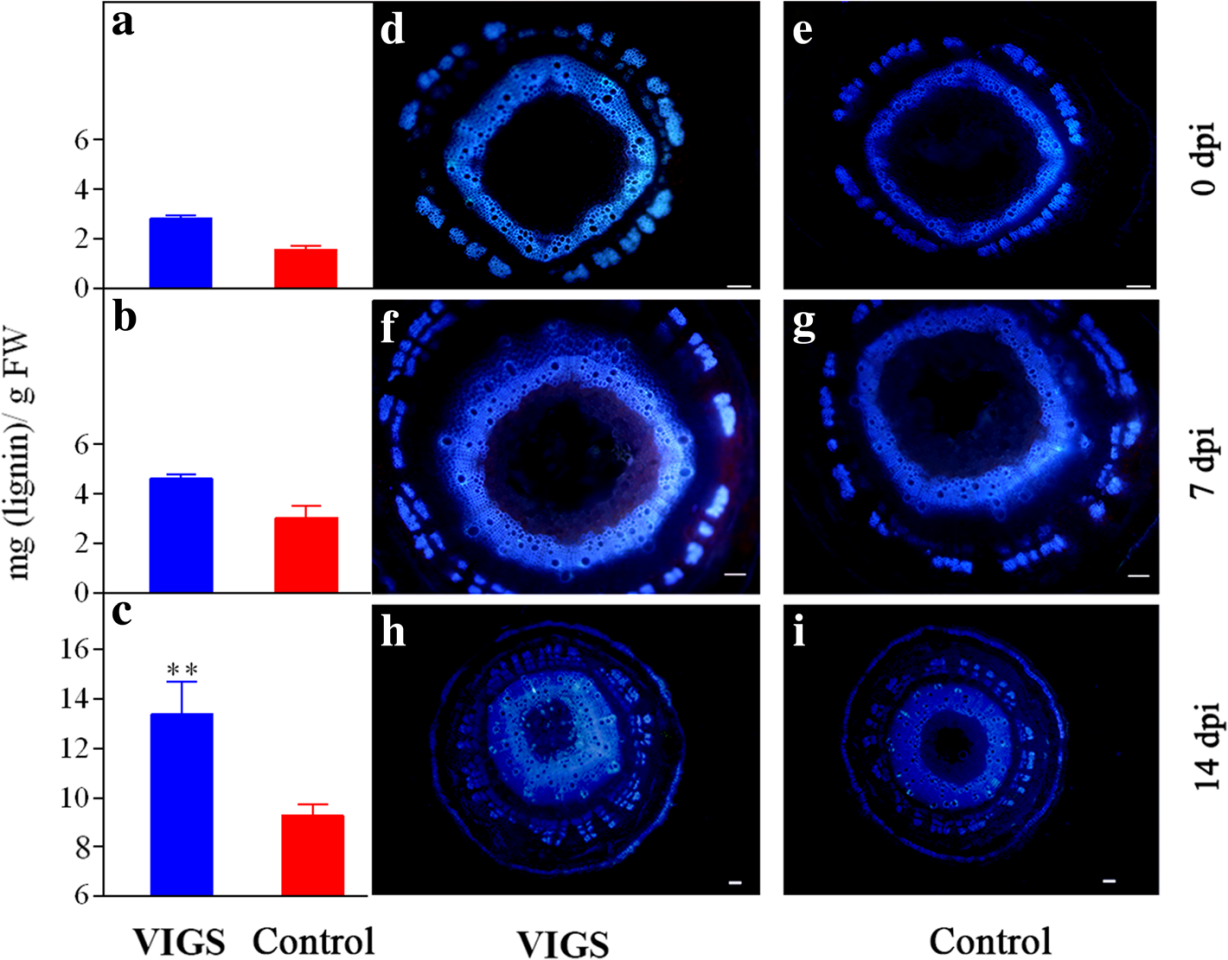
Analysis of lignin in stems of *HyPRP1*-silenced cotton plants after inoculation with *V. dahliae*. 10-day-old seedlings with two fully expanded cotyledons were used for VIGS. After two weeks later *HyPRP1*-silenced plants were inoculated with *V. dahliae*. Measurement and statistical analysis of the lignin content in cotton stems after inoculation with *V. dahliae* at 0 dpi (**a**), 7 dpi (**b**) and 14 dpi (**c**). At each time point of each treatment, three separate plants were examined. The results show means ± SE of values for three independent experiments. Asterisks indicate a statistically significant difference according to Sidak’s multiple comparisons test (**P < 0.01). Fluorescence microscopy of a 1-cm transverse stem section from control and *HyPRP1*-silenced cotton plants at 0 dpi (**d**&**e**), 7 dpi (**f**&**g**) and 14 dpi (**h**&**i**). Bar = 50 μm. Lignin autofluorescence was visualized following ultraviolet excitation at 365 nm

To further determine the effect of silenced *HyPRP1* on the cell wall, the cotton stems were histologically examined. 10-day-old seedlings with two fully expanded cotyledons were used for VIGS. After two weeks later *HyPRP1*-silenced plants were inoculated with *V. dahliae*. At 14 dpi, cross sections of the basal part of stems revealed that the thickness of interfascicular fiber walls increased obviously in the *HyPRP1*-silenced plants compared with the control (Fig. 6a and a′). Moreover, we examined vessel walls in ultra-thin sections using transmission electron microscopy, which revealed a dramatic reduction in thickness (Fig. 6b and b′). These results demonstrated that silencing of *HyPRP1* does not affect the development of the cell wall under a pathogen-free condition, but repression of *HyPRP1* expression significantly enhances cell wall thickening in response to *V. dahliae*. Further, lignin contents were estimated in cell wall residues. The data showed that *HyPRP1*-silenced plants had higher lignin content compared with the control at 0 dpi, 7 dpi and 14 dpi (Fig. 7a-c). Accordingly, the autofluorescence of lignified cell walls in the xylem and vascular bundles of *HyPRP1*-silenced plants was more intense and covered a greater area compared with the control (Fig. 7d-i).

### Silencing of *HyPRP1* enhanced ROS accumulation in root tips of cotton infected with *V. dahliae*

**Fig. 8:**
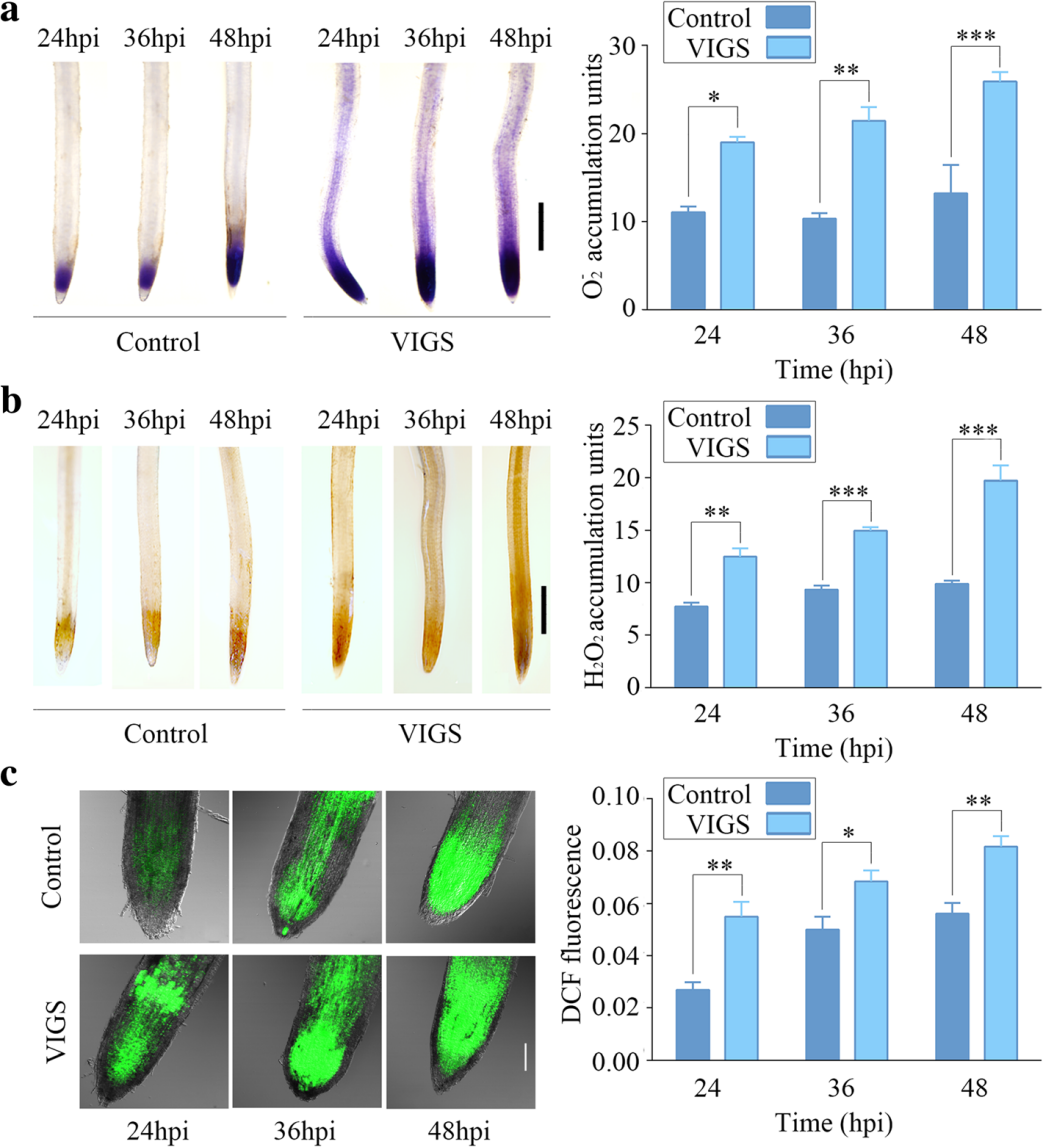
Comparison of ROS accumulation in root tips of *HyPRP1*-silenced cotton seedlings inoculated with *V. dahliae*. **a** Representative microphotographs of NBT staining. The roots were inoculated with NBT for 40 min and were then subjected to microscopic observation to determine O_2_^−^ production. Scale bar = 1 mm. **b** Representative microphotographs of DAB staining. The roots were placed in DAB for 10 h and were then subjected to microscopic observation to determine H_2_O_2_ production. Scale bar = 1 mm. **c** Representative confocal images of DCFH-DA staining. The roots were placed in DCFH-DA for 30 min and were then examined to determine ROS production. Scale bar = 100 μm. At each time point of each treatment, nine roots from three separate plants were examined. Data represent the means ± SE from three biological replicates; asterisks indicate statistically significant differences according to Tukey’s multiple comparisons test (*P < 0.05, **P < 0.01, ***P < 0.001). 10-day-old seedlings with two fully expanded cotyledons were used for VIGS. After two weeks later *HyPRP1*-silenced plants were inoculated with *V. dahliae*

We evaluated whether *HyPRP1* silencing would result in cotton generating more ROS in response to *V. dahliae* infection. DAB and NBT staining showed that *HyPRP1* VIGS cotton had significantly higher levels of O_2_^−^ and H_2_O_2_ compared to the control under *V. dahliae* infection (Fig. 8a and b). In particular, VIGS plants not only showed darker staining approximately one millimeter from the tip, but staining was also detected in the upper part of the root (Fig. 8a and b). DCFH-DA staining further confirmed enhanced ROS levels in *HyPRP1* VIGS cotton roots compared to the control (Fig. 8c). These results suggest that *HyPRP1* is involved in ROS production in cotton infected with *V. dahliae*.

## Discussion

### *HyPRP1* is a negative regulator in cotton resistance to Verticillium wilt

The roles of *HyPRPs* in response to multiple abiotic and biotic factors such as cold, salinity, drought and pathogens have been inferred primarily from their expression profiles [3, 8, 19–22]. Remarkably, all of these *HyPRP* genes have been reported to be up-regulated by abiotic factors and down-regulated upon infection with pathogens, e.g., *HyPRP1* in *C. annuum* and *N. benthamiana* following *P. capsici* infection [3] and *PvPRP1* in cell cultures of *P. vulgaris* treated with an elicitor [8]. Similarly, in our study, transcriptional suppression of *HyPRP1* in roots of both resistant and susceptible cotton was also detected after *V. dahliae* infection (Figs. 1d and 2b). This indicates that *HyPRP1* is involved in the interaction between cotton and Verticillium. Further, based on loss- and gain- of function studies comprising VIGS and over-expression in cotton and *Arabidopsis* plants, respectively, we showed that *HyPRP1* was negatively correlated with resistance to *V. dahliae* (Figs. 3 and 4). Thus, it is reasonable to speculate that HyPRP1 functions in the process of cotton resistance to Verticillium wilt as an important negative regulator.

### *HyPRP1* is potentially down-regulated by SA in cotton resistance against *V. dahliae*

Upon pathogen attack, plants can rapidly initiate an immune response that is regulated by specific phytohormones, which vary greatly in composition, quantity and timing [23, 24]. Through analysis of differential gene expression and transcription profiling of cotton inoculated with *V. dahliae*, SA-, ABA-, JA- and ET-mediated signaling pathways have been proven to contribute to *V. dahliae* resistance [14, 25]. ABRE (ABA-responsive element) (− 292), TCA-element and ERE (ethylene responsive element) (− 1128) have been reported in the upstream region of many genes that showed regulated expression in response to ABA, SA and ET, respectively [26–28]. In this paper, these potential *cis*-regulatory elements were also found in *pGbHyPRP1* (Fig. 5b). Furthermore, the expression profiles of *GbHyPRP1* following treatment with these phytohormones were examined. The expression of *GbHyPRP1* was significantly down-regulated by SA but up-regulated by ABA, JA and ET (Fig. 5c). Activation of complicated and concerted phytohormone signaling networks is an important regulatory mechanism of immunity employed by plants. In many cases, these hormones interact antagonistically or synergistically with each other [29]. Generally, pathogens that require a living host (biotrophs) are implicated in SA-mediated defense responses, whereas pathogens that kill the host and feed on the contents (necrotrophs) are associated with JA/ET-mediated defenses [23, 30]. Interestingly, however, *V. dahliae* is a hemibiotrophic phytopathogenic fungus. Thus, we infer that *HyPRP1* taking part in cotton resistance to *V. dahliae* is probably regulated by a complex phytohormone signaling network. The *HyPRP1* transcript levels showed quite similar down-regulation in SA-treated and *V. dahliae*-inoculated cotton seedlings (Figs. 1d, 2b and 5c). Additionally, *V. dahliae* infection significantly increased SA levels in *G. thurberi* [31], *G. hirsutum* and *G. barbadense* seedlings (our unpublished data). Thus, we speculate that *HyPRP1* may be mainly and negatively regulated by SA signaling in cotton resistance against *V. dahliae*.

### HyPRP1 participates in complex interactions within the cell wall polymer network in cotton infected with *V. dahliae*

Cell wall proteins are essential constituents of plant cell walls and are involved in modification of the cell wall structure [32]. Arabinogalactan protein 31 (AGP31), a remarkable plant cell-wall protein, comprises an SP, a short AGP domain, an His-stretch, a PRD and a PAC (PRP-AGP containing Cys) domain [33–35]. Arabidopsis AGP31 is able to bind methylesterified polygalacturonic acid, possibly through its His-stretch, and to interact with itself in vitro through its PAC domain [34]. Similarly, cotton HyPRP1 also contains an SP, a basic histidine-rich domain embedded within the PRD and a cysteine-containing domain embedded within the Pollen Ole e I domain (Fig. 1a and Additional file 1: Figure S1). The cell wall, a physical barrier that pathogens need to breach to colonize the host plant, is typically reinforced with the phenolic polymer lignin [36, 37]. Lignin is believed to play a critical role in the resistance of cotton to *V. dahliae* [13]. By comparative transcriptome analysis of *HyPRP1*-silenced cotton plants and the control inoculated with *V. dahliae*, 79 DEGs potentially involved in cell wall biogenesis were identified (Table 2). Of these genes, *Gh_D13G1445* was described as a Mannose-1-phosphate guanylyltransferase catalyzing the production of GDP-mannose. Arabidopsis *cyt1* (encodes mannose-1-phosphate guanylyltransferase) mutant cause changes in the cell wall composition, such as dramatic decrease in cellulose content [38]. UDP-glycosyltransferases (UGTs) can influence the resistance of plants to infection by pathogenic microorganisms through regulating the glycosylation of phenylpropanoid and phenylpropanoid-derived compounds, which are essential for the synthesis of lignin [39]. The xyloglucan endotransglucosylase/hydrolases (XTHs), specifically hydrolyzing xyloglucan as a substrate, are considered to be involved in the construction and restructuring of xyloglucan cross-link in plant cell wall [40]. Calmodulin, a highly conserved Ca^2+^-binding protein, acts as an intermediary connecting Ca^2+^ signals involved in plant defence reactions [41]. Most of DEGs mentioned above have higher transcript levels in *HyPRP1*-silenced plants, suggesting that *HyPRP1* is a negative regulator of cell wall-related genes. Further, we observed a dramatic thickening of interfascicular fiber walls and vessel walls (Fig. 6) and an increase in lignin (Fig. 7) in the *HyPRP1*-silenced cotton plants compared with the control after inoculation with *V. dahliae*. On the other hand, some DEGs were down-regulated, which seems to be a negative impact on cell-wall thickening and lignin accumulation. For example, phenylalanine ammonia-lyase (PAL) is the first committed enzyme in the phenylpropanoid biosynthesis, which engenders a variety of precursors of important secondary metabolites, mainly including flavonol glycosides and lignin. In our study, *PAL2* (*Gh_D06G0758*) was shown to be significantly downregulated. We inferred that *PAL* transcription was feedback regulated by particular biosynthetic intermediates [42], which means that the expression of *PAL* may not be always positive correlation with lignin accumulation. Although the roles of many cell wall proteins have been studied broadly, the knowledge on the interaction between components is lacking. Thus, we speculated that *HyPRP1* participates in complex interactions within the cell wall polymer network in cotton infected with *V. dahliae*.

### HyPRP1 contributes to Veticillum defense by enhancing ROS accumulation

ROS have been studied extensively for their roles in interactions between plants and foliar pathogens. Little is known about ROS synthesis and function in defense reactions of the root, but ROS are consistently observed to accumulate in a plant after the perception of pathogens [18]. In the Verticillium-cotton interaction, the generation of H_2_O_2_ was observed in the roots of cotton infected with *V. dahliae* [43]. Moreover, transgenic tomato plants expressing the *Ve* resistance gene accumulated H_2_O_2_ upon *V. dahliae* infection [44]. Likewise, transgenic cotton plants expressing a fungal endochitinase gene were more resistant and accumulated ROS faster than the control following pathogen inoculation [45]. These results indicate that cotton plants infected with *V. dahliae* are accompanied by increased ROS accumulation. In our study, silencing of *HyPRP1* markedly enhanced ROS accumulation in the root tips (Fig. 8). Therefore, we reasonably inferred that cotton negatively modulates *HyPRP1* transcription to generate ROS used for defense against Veticillum. Further studies are needed to elucidate the roles of ROS in cotton resistance to Verticillium. However, we suggest that ROS perform three possible functions: (i) ROS are primary immune signaling molecules [46]; (ii) ROS mediate cell wall modifications [47]; and (iii) ROS are important modulators that play a role in defense-related protein post-translational modifications [48].

## Conclusions

Based on our research and existing developments on how plants resist Verticillium wilt, we propose a model of *HyPRP1*-mediated cotton defense against *V. dahliae*. Upon *V. dahliae* attack, recognition by cotton plants results in the activation of immune responses, including the production of a specific combination of the signals such as SA and JA, which have been proved to be involved in the plant-*V. dahliae* interaction [14, 25]. The expression of *HyPRP1* is most likely and mainly down-regulated by SA signaling. A significant reduction in HyPRP1 may affect the cell wall polymer network, including an increase in the thickness of the cell wall and the content of lignin required to prevent *V. dahliae* infection. Alternatively, down-regulation of *HyPRP1* obviously enhances the accumulation of ROS, which could mediate the establishment of a cotton defensive response to *V. dahliae*.

## Methods

### Plant materials and growth conditions

The cotton seeds *G. barbadense* cv. Pima90–53, *G. hirsutum* cv. CCRI8 and ND601 were preserved at the North China Key Laboratory for Crop Germplasm Resources of Education Ministry, Hebei Agricultural University, Baoding, China. For transcriptional analysis of *HyPRP1* in different tissues and response to *V. dahliae* by qPCR, two-week-old cotton seedlings were cultivated on Murashige and Skoog (MS) medium and were inoculated with *V. dahliae* as described by Zhang et al. [14]. For VIGS and the hormone treatment experiment, seeds were sterilized in 20% (*V*/V) commercial bleach (the final concentration of sodium hypochlorite was approximately 1%) for 20 min followed by washing four times with distilled water. Seeds were soaked in distilled water for 2 days and then germinated on wet towels for another 2 days at 25 °C. Germinant seeds were transferred to pots containing commercially sterilized soil (a mixture of soil, peat, and composted pine bark) and covered with a plastic dome in a growth room at 25 °C under a 14-h light/10-h dark cycle. The *A. thaliana* accession Columbia was grown in commercially sterilized soil at 22 °C, 70% relative humidity, and ~ 150 μE m^− 2^ s^− 1^ under a 9-h photoperiod.

### *V. dahliae* cultivation

A highly aggressive defoliating fungus, *V. dahliae* strain Linxi2–1, was isolated from a symptomatic upland cotton plants growing in agricultural fields near Linxi, Hebei Province, China, and preserved in North China Key Laboratory for Crop Germplasm Resources of Education Ministry, Hebei Agricultural University. *V. dahliae* was cultivated on potato dextrose agar (PDA) plates for 10 d and then inoculated into Czapek’s broth on a shaker at 150 rpm for 1 week at 25 °C in the dark. Spores were harvested by filtration through folded Fisherbrand™ lens paper and were resuspended in sterile distilled water to a specific density.

### Cloning of *HyPRP1* and the *GbHyPRP1* promoter region

*GbHyPRP1* was identified from a full-length cDNA library of *G. barbadense* Pima90–53 [14]. The full-length cDNA of *HyPRP1* from other upland cotton cultivars was obtained by homology-based cloning. The genomic walking method was performed to amplify its 5′ flanking (promoter) region using the Genome Walking Kit (Takara, Dalian, China) according to the manufacturer’s instructions. Nested sequence-specific primers designed on the basis of the known *GbHyPRP1* gene sequence and shorter arbitrary degenerate primers were chemically synthesized or provided by the kit. The HyPRP1 protein sequences were identified using NCBI Web BLAST (http://www.ncbi.nlm.nih.gov/) and were aligned using the Clustal W program (http://www.clustal.org/). The promoter sequence was analyzed using the software programs PlantCARE and PLACE to define putative *cis*-elements or binding sites for transcription factors [49, 50].

### qPCR and semi-RT-qPCR analysis

Total RNA was extracted using TRIzol® reagent (Invitrogen, Carlsbad, CA, USA) according to the manufacturer’s instructions. Each treatment, imposed on three pooled root, stem or leaf samples, was repeated at least three times in all experiments. RNA was quantified using a NanoDrop™ 1000 Spectrophotomete (Thermo Fisher Scientific). Subsequently, first-strand cDNA was synthesized from 1 μg of total RNA using the PrimeScript™ RT Reagent Kit with gDNA Eraser (TaKaRa, Dalian, China). qPCR was performed using a CFX96 Real-Time PCR Detection System (Bio-Rad, Hercules, CA, USA) and SYBR® Green reagent (TaKaRa, Dalian, China) as the reporter dye. Data were collected using CFX Manager™ software (Bio-Rad, Hercules, CA, USA). Target gene relative expression was normalized using *PP2A1* (catalytic subunit of protein phosphatase 2A) [51]. For semi-RT-qPCR, the reactions were run with a denaturation step of 95 °C for 5 min, followed by 25 cycles of 94 °C for 1 min, 55 °C for 30 s, 72 °C for 1 min, with a final extension at 72 °C for 10 min. PCR products were electrophoresed on 1% agarose gels.

### Subcellular localization analysis of transiently expressed fusion proteins

For subcellular localization studies, the *GbHyPRP1* ORF was amplified using PCR. The resulting product was inserted into the vector pDONR™207. Subsequently, the fragment was recombined into the destination vector pK7WGF2 [52] using L/R-Clonase. The construct was verified by sequencing and was transferred to the *Agrobacterium tumefaciens* GV3101 strain using the freeze/thaw method [53]. Transient transformation of tobacco leaf epidermal cells was performed as described in [54]. Localization of fluorescent proteins was monitored 3 days after infiltration using a confocal laser scanning microscope (FluoView FV1000; Olympus). A pCAMBIA derivative (pCamE) carrying a cauliflower mosaic virus 35S–driven GFP was used as the control [55].

### Hormone treatments

Cotton seedlings at the 2-cotyledon stage were sprayed with 100 μM SA, ABA, JA or ET and were covered with plastic bags to maintain 100% humidity. Cotyledon tissues were collected from hormone-treated plants at 6, 12, 24, 36 and 48 hps, immediately frozen in liquid nitrogen and then stored at − 80 °C until RNA extraction. The control seedlings were sprayed with distilled water.

### Generation of transgenic Arabidopsis lines

The coding sequence of *GbHyPRP1* was amplified from *G. barbadense* Pima90–53 cDNA and cloned into a pBI121 vector. A genomic *GbHyPRP1* upstream fragment was amplified and cloned into the pBI121 vector containing beta-glucuronidase (GUS) gene coding sequences, where the 35S promoter region was excised by digestion using *Pst*I and *Bam*HI restriction enzymes and was replaced with the sequence of the *GbHyPRP1* upstream fragment. The recombinant plasmid was transformed into *A. thaliana* Columbia wild type (WT) plants through *Agrobacterium tumefaciens* strain GV3101-mediated plant transformation using the floral dip method [56]. Primary transformants were selected for survival on ½ MS medium with 50 μg ml^− 1^ kanamycin. Homozygous plants were isolated using gene-specific primers. Semi-RT-qPCR was then performed on cDNA from overexpression lines to confirm the status of transcription using the full-length *GbHyPRP1* primers. Expression was normalized to the expression of actin. T3 transgenic *A. thaliana* plants carrying the *GbHyPRP1* promoter were used in histochemical assays for GUS staining as described by [57].

### VIGS assays in cotton

pTRV1 and pTRV2 from the Yule Liu research group at Tsinghua University (China) were used for the VIGS assays [58]. *HyPRP1* fragments (364 bp) were amplified (Additional file 5: Table S1) and inserted into the pTRV2 vector to generate the derivative pTRV-HyPRP1, which were transformed into *A. tumefaciens* strain GV3101. An *Agrobacterium*-mediated VIGS assay in cotton was performed as previously described [59]. Cotton seedings with two fully expanded cotyledons were utilized. At this stage, the true leaves had not yet emerged. The cloroplastos alterados 1 gene (CLA1) was used as a marker to monitor the silencing efficiency. The seedlings injected with *Agrobacterium* cultures harboring the pTRV1 and pTRV2 (empty vector) were used as control. VIGS assays were repeated at least three times using more than 30 plants from each treatment per repeat.

Twenty-four-day-old cotton seedlings were subjected to *V. dahliae* inoculation by root dipping in a spore suspension (10^7^ spores ml^− 1^) for 2 min and were then returned to their original pots. Four-week-old *Arabidopsis* plants were infected with *V. dahliae* by soil drenching using a 10-ml conidial suspension (10^6^ spores ml^− 1^) per pot (80 ml). Control plants were inoculated with distilled water in the same way. Cotton and *Arabidopsis* symptoms and disease index (DI) were scored at 15 dpi. The DI was calculated based on five disease grades as described previously [60]. Thirty-five plants were used per treatment, and each treatment was repeated three times. Plant resistance to *V. dahliae* was determined based on the DI, where > 35 = susceptible, 20 to 35 = tolerant, and 10 to 20 = resistant (National Standards of the People’s Republic of China GBT 22101.5–2009, Technical Specification for Evaluating Resistance of Cotton to Disease and Insect Pests - Part 5: Verticillium wilt).

### Primers

All primers used in this paper are listed in Additional file 5: Table S1.

### RNA-Seq library construction and analysis of transcriptome sequencing data

Two independent sets from both control and *HyPRP1*-silenced plants were sampled to generate two biological replicas. For each sample, the first true leaves from at least six plants were collected at 12 hpi and pooled to minimize plant-to-plant variation. Then, total RNA was extracted using TRIzol® reagent following the manufacturer’s instructions, after which genomic DNA was removed using DNase I (Invitrogen, Carlsbad, CA, USA). RNA purity was checked using a NanoPhotometer® spectrophotometer (IMPLEN, CA, USA), and concentration was measured in a Qubit® 2.0 Flurometer using the Qubit® RNA Assay Kit (Life Technologies, CA, USA). RNA integrity was assessed using the RNA Nano 6000 Assay Kit of the Bioanalyzer 2100 system (Agilent Technologies, CA, USA). Transcriptome sequencing was performed using an Illumina HiSeqTM 2000 sequencer (Illumina, San Diego, CA, USA).

Sequence tag preprocessing was performed according to a previously described protocol [61]. Paired-end clean reads were aligned to the reference genome of *G. hirsutum* L. acc. TM-1 (https://www.cottongen.org/) using Bowtie v2.0.6 and TopHat v2.0.9. Differential expression analysis of *HyPRP1*-silenced and unsilenced (mock) groups (two biological replicates per group) was performed using the DESeq R package (1.10.1). The resulting *P*-values were adjusted using Benjamini and Hochberg’s approach for controlling the false discovery rate. Genes with an adjusted *P* < 0.05 according to DESeq were assigned as differentially expressed.

### Examination of cell walls

Stems were cut into pieces (1 mm^3^) and fixed in 2.5% (wt/vol) glutaraldehyde in 0.1 M phosphate buffer, postfixed in 1% osmium tetroxide, and then embedded in Spurr’s resin. Semi-thin sections (1 μm), prepared using a Leica UC6 (Leica, Illinois, USA), were hot-stained with a 1% toluidine blue water solution. Images were captured using a digital camera (Digital sight DS-L1, Nikon, Japan) attached to a Olympus BX51 microscope (Tokyo, Japan). Ultra-thin sections (70 nm thick), prepared using a Leica Ultracut R ultramicrotome (Leica, Illinois, USA), were stained with uranyl acetate and lead citrate and examined under a JEM-1230 electron microscope (JEOL Ltd., Tokyo, Japan). Approximately 60 cells were measured per sample to determine the thickness of the cell walls.

### Lignin extraction and quantification

Stem samples were collected from both HyPRP1-silenced and control plants at 0, 7 and 14 dpi*.* Three samples were collected per plant at different time intervals; the samples were crushed immediately in liquid nitrogen and freeze-dried. The extraction and quantification of lignin in cell walls were performed according to the method of Schenk et al., 2014 [62].

### Identifiction of lignin deposition in cell walls using histochemistry and autofluorescence

Standard transverse free-hand sections were collected from the stems of cotton seedlings (5 cm from the stem base). Lignin autofluorescence was imaged using a BX51 microscope with both UV excitation and brightfield modes.

### Biological analysis of ROS accumulation

Since ROS include various forms of reduced and chemically reactive molecules such as hydrogen peroxide (H_2_O_2_) and the superoxide oxygen anion (O_2_^−^), we used NBT and DAB dyes, as well as the ROS-reactive fluorescent probe DCFH-DA, to detect the accumulation of ROS. Yellow, water-soluble NBT can be reduced by O_2_^−^ to blue, water-insoluble formazan. DAB polymerizes at sites of peroxidase activity into a reddish brown polymer in the presence of H_2_O_2_. Fluorescence-free DCFH-DA can cross the cell membrane and, after hydrolyzation, be oxidized by ROS to form highly fluorescent DCF [18]. ROS (H_2_O_2_) accumulation was visualized by incubating intact cotton roots in 5 μM DCFH-DA (dichlorofluorescein diacetate; Beyotime, Nanjing, China) for 20 min at 37 °C in the dark or in 10 mM K-citrate buffer (pH 6.0) containing 2.5 mM DAB (3,3′-diaminobenzidine) for 12 h, as described by [63]. ROS (O_2_^−^) production was also visualized by incubating intact roots in 10 mM K-citrate buffer (pH 6.0) containing 0.5 mM NBT (nitrotetrazolium blue chloride) for 30 min at 37 °C. An Olympus FV1000 laser confocal microscope with an excitation wavelength of 488 nm was used for the analysis of DCF fluorescence. For DAB and NBT staining, the roots were boiled in 95% ethanol for 10 min and then rinsed twice with 50% ethanol, after which images were captured under a light microscope (BX51; Olympus, Tokyo, Japan) using a digital camera (DP71; Olympus, Tokyo, Japan) and Image-Pro® Plus 6.0 software (Media Cybernetics, Rockville, MD, USA). Quantization of fluorescence and coloration intensity was performed using ImageJ software version 1.48 (National Institute of Mental Health, Bethesda, MD, USA).

### Statistical analysis

All experiments were repeated at least three times for each determination. Statistical analysis of data was conducted with the software of GraphPad Prism® 6 (Graph Pad, San Diego, CA, USA). The *P*-value less than 0.05 was considered to be statistically significant.

## Additional files

Additional file 1: **Figure S1.** Alignment of the amino acid sequences of Sea Island cotton Pima90–53 HyPRP1 with those of six other upland cotton cultivars including TM-1, Coker312, ND601, CCRI8, JiMian20, and NongDaMian7. The alignment results showed that HyPRP1 shares a significant degree of sequence identity in cotton. (TIF 615 kb)

Additional file 2: **Figure S2.** A DNA fragment upstream of the *GbHyPRP1* coding sequence was isolated and then designated as p*GbHyPRP1*, which is 1431 bp in length and contains 41 nucleotides of the 5′ -terminal regions of the *GbHyPRP1* cDNA. The first base of the cDNA was designated as the putative transcription start site (+ 1). (TIF 2613 kb)

Additional file 3: **Figure S3.** At approximately two weeks post *Agrobacterium* infiltration, the leaves of *CLA1*-VIGS plants started to displayed the albino phenotype on the true leaves (A). At the same time, the silencing of *HyPRP1* gene expression in VIGS and control plants was confirmed by semi-RT-qPCR analysis (B). (TIF 9955 kb)

Additional file 4: **Data S1.** DEGs in V12 (VIGS at 12 hpi) versus C12 (Control at 12 hpi) using Padj (adjusted P) value < 0.05 as criteria. (XLSX 106 kb)

Additional file 5: **Table S1.** Primers used in this study. (DOCX 15 kb)

## Abbreviations

ABA: Abscisic acid
CLA1: Cloroplastos alterados 1
DEGs: Differentially expressed genes
DI: Disease index
ET: Ethylene
GUS: beta-glucuronidase
HyPRP: Hybrid proline-rich protein
JA: Jasmonic acid
ORF: Open reading frame
PDA: Potato dextrose agar
ROS: Reactive oxygen species
SA: Salicylic acid
VIGS: Virus-induced gene silencing
WT: Wild type
