# Supplementary figures and images for: HyPRP1 performs a role in negatively regulating cotton resistance to V. dahliae via the thickening of cell walls and ROS accumulation
Source: BMC Plant Biol. 2018 Dec 7;18:339. doi: 10.1186/s12870-018-1565-1 (PMC6286592; doi:10.1186/s12870-018-1565-1)

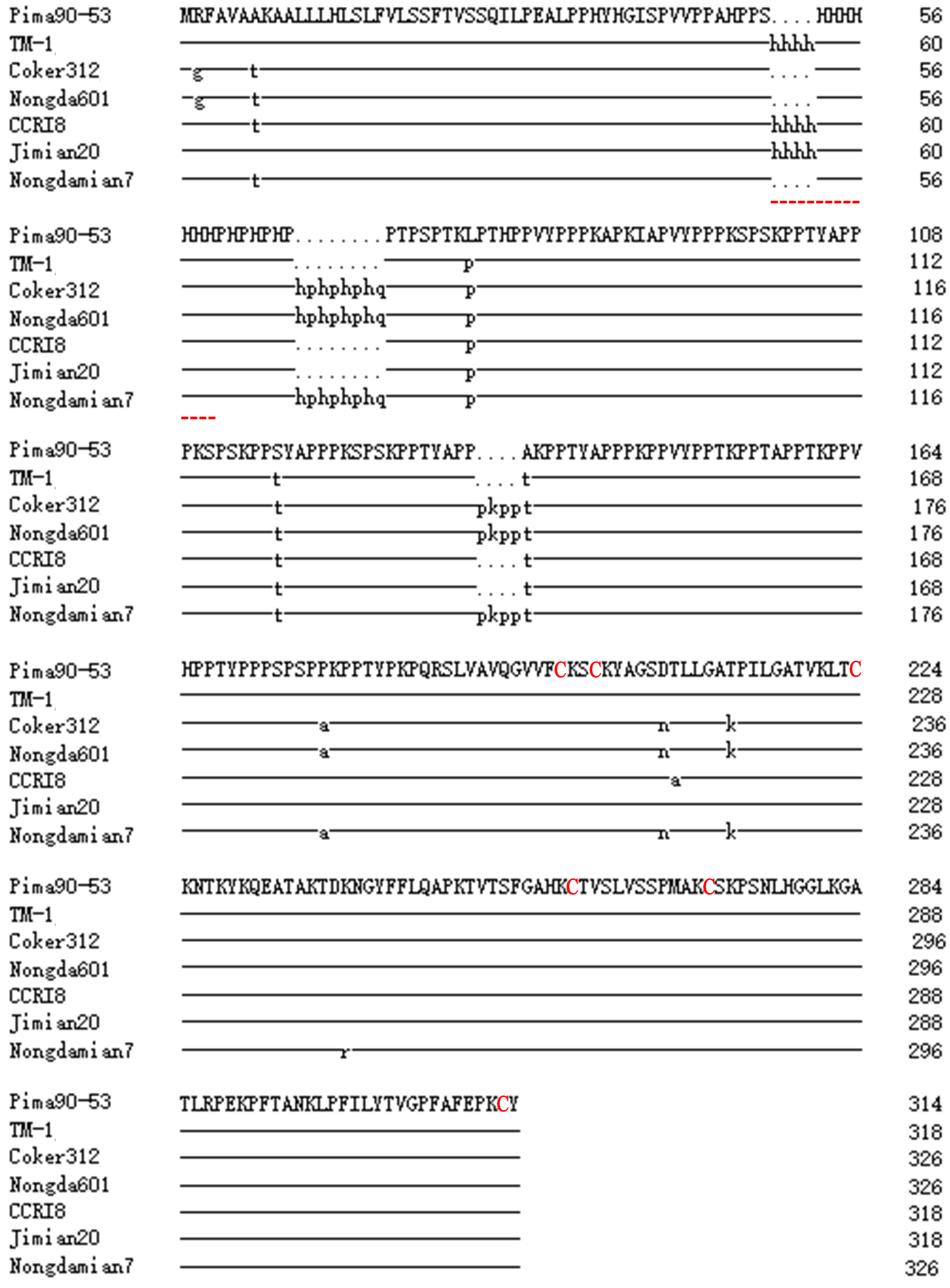

Supplement: Supplementary file 1 — Figure S1. Alignment of the amino acid sequences of Sea Island cotton Pima90–53 HyPRP1 with those of six other upland cotton cultivars including TM-1, Coker312, ND601, CCRI8, JiMian20, and NongDaMian7. The alignment results showed that HyPRP1 shares a significant degree of sequence identity in cotton. (TIF 615 kb) [file 12870_2018_1565_MOESM1_ESM.tif]

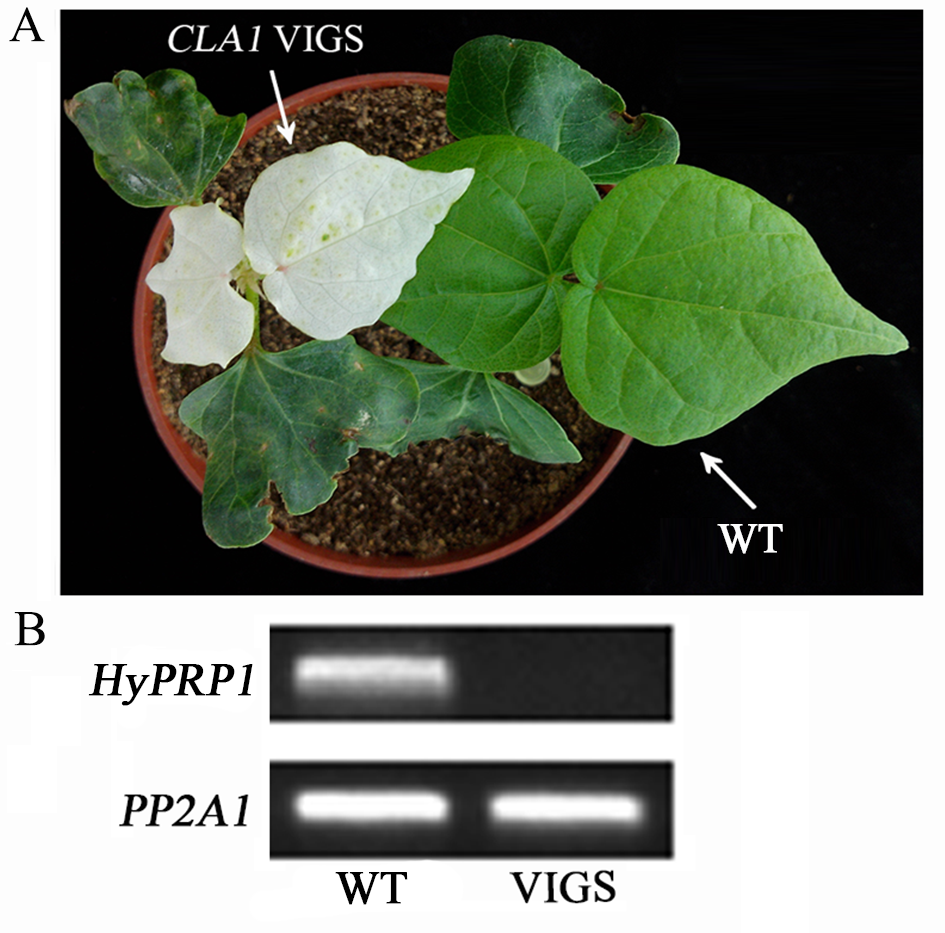

Supplement: Supplementary file 2 — Figure S2. A DNA fragment upstream of the GbHyPRP1 coding sequence was isolated and then designated as pGbHyPRP1, which is 1431 bp in length and contains 41 nucleotides of the 5′ -terminal regions of the GbHyPRP1 cDNA. The first base of the cDNA was designated as the putative transcription start site (+ 1). (TIF 2613 kb) [file 12870_2018_1565_MOESM2_ESM.tif]

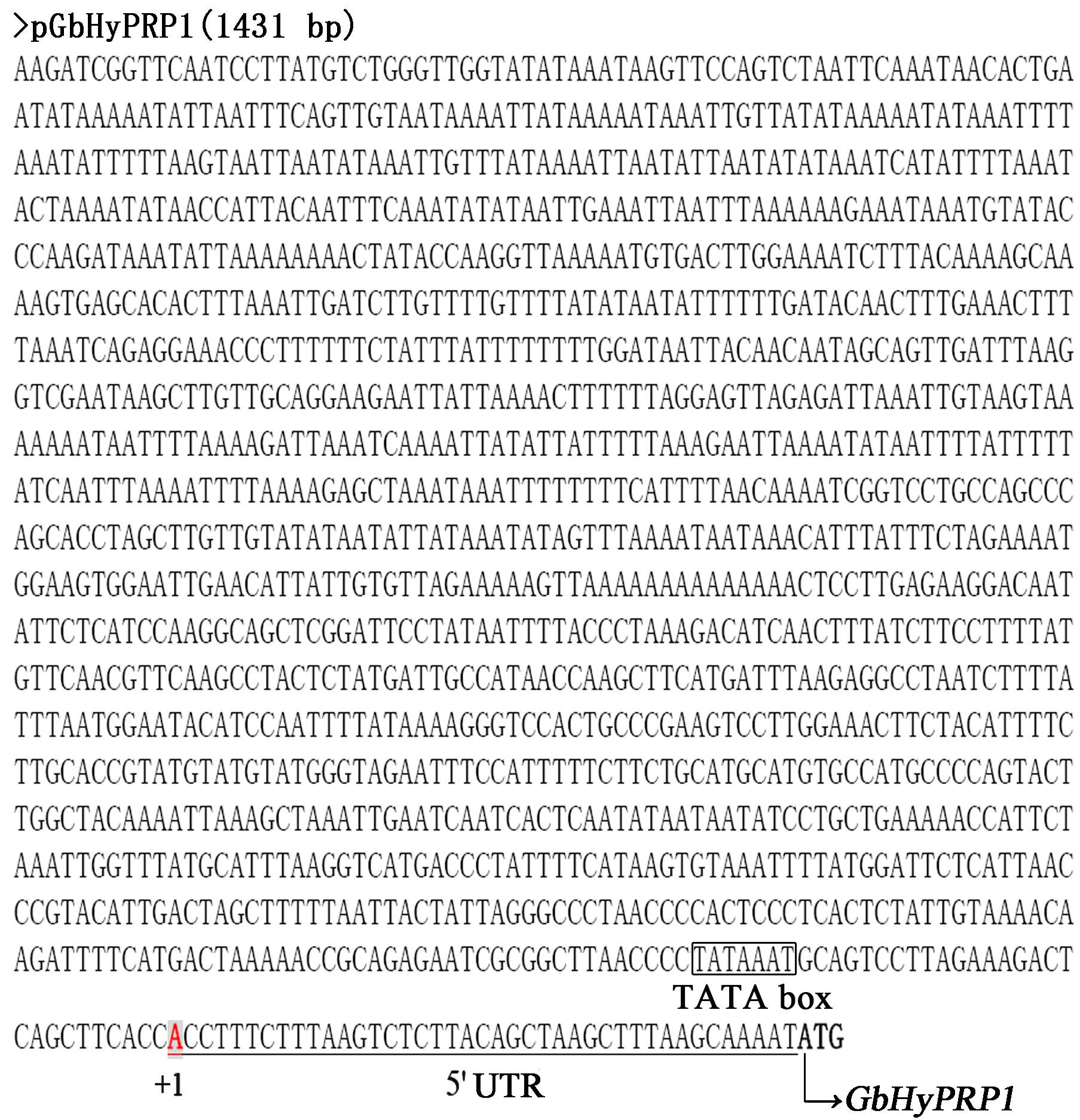

Supplement: Supplementary file 3 — Figure S3. At approximately two weeks post Agrobacterium infiltration, the leaves of CLA1-VIGS plants started to displayed the albino phenotype on the true leaves (A). At the same time, the silencing of HyPRP1 gene expression in VIGS and control plants was confirmed by semi-RT-qPCR analysis (B). (TIF 9955 kb) [file 12870_2018_1565_MOESM3_ESM.tif]
