# Supplementary material for: HyPRP1 performs a role in negatively regulating cotton resistance to V. dahliae via the thickening of cell walls and ROS accumulation
Source: BMC Plant Biol. 2018 Dec 7;18:339. doi: 10.1186/s12870-018-1565-1 (PMC6286592; doi:10.1186/s12870-018-1565-1)
Supplement: Supplementary file 5 — Table S1. Primers used in this study. (DOCX 15 kb) [file 12870_2018_1565_MOESM5_ESM.docx]

| Primer name | Primer sequence (5' to 3') | Use and specification |
| --- | --- | --- |
| GbHy-F  GbHy-R | ATGAGGTTTGCAGTAGCAGC  CTAGTAGCATTTGGGTTCG | For cloning of *GbHyPRP1* from  *G. barbadense* Pima90-53 |
| Hy-F  Hy-R | ATGRGGTTTGCAGTAGCAGC  CTAGTAGCATTTGGGTTCG | Homology cloning of HyPRP1 from other upland cotton cultivars |
| GSP1  GSP2 | TAACTTGGCGGCTTGCTGGGTGACTTGG  GCGTAAGTTGGCGGCTTGCTTGGTGAC | For amplifying  *GbHyPRP1* 5’ flanking (promoter) region |
| 35S-F  35S-R | GGATCCATGAGGTTTGCAGTAGCAGC  GAGCTCCTAGTAGCATTTGGGTTCG | For cloning of *GbHyPRP1* in pBI121 vector and positive selection of transgene |
| GbHy-PstI  GbHy-BamHI | CTGCAGAAGATCGGTTCAATCCTTATG  GGATCCATTTTGCTTAAAGCTTAGCTG | For cloning of *GbHyPRP1* promoter in pBI121 vector |
| Actin-F  Actin-R | TCCCTCAGCACATTCCAGCAGAT  AACGATTCCTGGACCTGCCTCATC | Housekeeping genes of *A. thaliana* (At3g18780) for Semi-RT-qPCR |
| PP2A1-F PP2A1-R | GATCCTTGTGGAGGAGTGGA  GCGAAACAGTTCGACGAGAT | For RT-qPCR analysis of HyPRP1 in cotton |
| HyPRP1-RT-F  HyPRP1-RT-R | CAGCAAGCCGCCAAGTTAC  GCGATGGTGGAGGATATGTTG | For RT-qPCR analysis of HyPRP1 in cotton |
| TRV-F  TRV-R | GAATTCGCACCCAAGATTGCTCCAGTTTA  GGTACCCAGATCCAGCGTATTTGCATGACT | For cloning of GhHyPRP1 in pTRV2 vector |
| CLA1-F  CLA1-R | GAATTCGCCCTTTGTGCATCTTC  GGTACCCTCTAGGGGCATTGAAG | For cloning of GhCLA1 in pTRV2 vector |

Table S1. Primers list

Restriction sites are underlined
